# An “Engram-Centric” Approach to Transient Global Amnesia (TGA) and Other Acute-Onset Amnesias

**DOI:** 10.3390/neurolint17010008

**Published:** 2025-01-15

**Authors:** Andrew J. Larner

**Affiliations:** Department of Brain Repair & Rehabilitation, Institute of Neurology, University College London, London WC1E 6BT, UK; andrew.larner2@nhs.net

**Keywords:** amnesia, ecphory, engram, functional amnesia, TEA, TGA

## Abstract

The differential diagnosis of acute-onset amnesia includes transient global amnesia (TGA), transient epileptic amnesia (TEA), and functional (or psychogenic) amnesia. The most common of these, TGA, is a rare but well-described condition characterised by a self-limited episode of dense anterograde amnesia with variable retrograde amnesia. Although the clinical phenomenology of TGA is well described, its pathogenesis is not currently understood, thus preventing the development of evidence-based therapeutic recommendations. Here, TGA, TEA, and functional amnesia are considered in light of the historical engram conception of memory, now informed by recent experimental research, as disturbances in distributed ensembles of engram neurones active during memory formation and recall. This analysis affords therapeutic implications for these conditions, should interventions to reactivate latent or silent engrams become available.

## 1. Introduction: Transient Global Amnesia (TGA)

Towards the end of November 1957, a 55-year-old school teacher, Mr. H., and his wife travelled from their home in Brooklyn to meet their daughter in a department store. During their meeting, he excused himself and about 35 min later was found apparently confused and bewildered, asking where he was and where he was going. His daughter, a physician, observed that her father was repeatedly asking the same questions and had a loss of memory for recent events; despite this, his neurological examination was normal. About five hours after the onset of symptoms, an electroencephalograph (EEG) was performed, which was normal. By the evening his memory was starting to return, and on the following day it was normal aside from a memory gap relating to the hours when he was confused.

The case of Mr. H. was subsequently presented to the New York Neurological Society at the New York Academy of Medicine on 9 October 1958 by Dr. Morris B. Bender (1905–1983) [1]. Bender, Neurologist to the Mount Sinai Hospital in New York City, titled his presentation “Single episode of confusion with amnesia”, as for similar cases he had previously reported [2]. However, by the time of Bender’s presentation to the New York Neurological Society, C. Miller Fisher (1913–2012) and Raymond D. Adams (1911–2008) in Boston had described similar cases using the term “transient global amnesia” (TGA) [3]. The latter terminology has persisted [4], in part because Bender erred in thinking such cases consisted of single, isolated, episodes (even though the amnesia is not “global” in the context of the current understanding of memory function). Bender’s 1960 publication described 26 patients, none of whom had had a second event recorded in up to ten years of follow-up. However, patients with recurrent episodes of TGA were later described, although this was initially thought unusual [5,6].

TGA has intrigued clinicians over the subsequent decades since these descriptions. Large amounts of empirical data have been collected regarding clinical features, investigation findings, prognosis, and epidemiology, including predisposing and precipitating factors [7,8]. Whilst certain factors appear to increase the likelihood of having TGA, including demographic (e.g., age), environmental (e.g., water contact, temperature change), or lifestyle (e.g., sexual activity) factors, these are neither necessary nor sufficient for TGA to occur. Furthermore, the pathogenesis of such relatively stereotyped and apparently self-limiting episodes remains unknown. Aetiological speculations, including a type of epileptic seizure [4], a cerebrovascular event [9], and a form of migraine, tell us little, if anything, about the pathophysiology of TGA. Hence, no evidence-based therapeutic or prophylactic recommendations can currently be made [8]. Nevertheless, it is recognised that TGA may tell us something about the cognitive architecture of the brain and the mechanisms of human memory.

## 2. Differential Diagnosis of Acute-Onset Amnesia

The differential diagnosis of acute-onset amnesia is potentially broad, including certain focal cerebrovascular accidents (“strategic stroke”), post-traumatic amnesia, and toxic or drug-related amnesia [10]. Only two specific differentials will be considered here: transient epileptic amnesia and functional amnesia.

There is no evidence that the great 19th-century clinical neurologist John Hughlings Jackson (1835–1911) encountered examples of TGA; the earliest clinical description recognised hitherto dates from 1909, with no compelling account dating from the 19th century [11,12]. However, Jackson was certainly familiar with amnesia in the context of epileptic seizures—for example, in the case of his patient named “Z”, himself a doctor, who gave an account of his amnesia associated with some of his seizures [13]. These later proved to be associated with a focal lesion in the temporal lobe [14], such that “Z” may perhaps represent the first reported or index case of what subsequently came to be called transient epileptic amnesia (TEA) [15,16].

TEA is now recognised to be an important differential diagnosis of TGA. TEA episodes differ clinically from TGA in that they are generally briefer, less than 1 h, and recurrent, often emerging on awakening from sleep. About half of patients have a dense ictal amnesia with repetitive questioning, but the remainder may have partial recall of the episode, in that they are able to “remember not being able to remember”. Prescription of anti-seizure medications often results in remission of TEA episodes. However, interictal assessment of memory shows autobiographical amnesia, accelerated long-term forgetting, and topographical amnesia in around three-quarters of patients [17].

Another amnesic syndrome that enters the differential diagnosis of acute-onset amnesia is variously known as psychogenic amnesia, transient psychological amnesia, mnestic block syndrome, dissociative amnesia, or the currently favoured terminology, functional amnesia [18]. Sometimes triggered by psychological stress or a minor head injury, the clinical picture in functional amnesia is typically characterised by dense retrograde amnesia mainly affecting the episodic–autobiographical domain of memory, sometimes of such density that heavily over-learned material such as personal identity and personal semantic memory may be apparently lost. However, unlike TGA and TEA, there is generally no anterograde amnesia, as the ability to learn new information is usually preserved. Furthermore, although disability may be long-lasting, the apparently spontaneous recovery of “lost” memories may occur, partially or totally, after variable periods of time [19]. There are no evidence-based recommended treatment options in functional amnesia; indeed, no controlled clinical trials have been reported.

Had Hughlings Jackson learned of TGA and functional amnesia, how might he have characterised them? He conceived of epileptic seizures as an example of dissolution of the nervous system, a failure (negative state) of the function of higher centres with loss of their inhibitory control over lower centres resulting in the abnormal mental state of amnesia (positive state). Presumably, he might have thought of TGA in a similar way. To use Jackson’s pathophysiological framework, the negative features of TGA and of functional amnesia might be conceived to reflect non-functioning rather than destroyed tissue [20]. Even in the case of destroyed tissue, Jackson had a “theory”, which he termed “a doctrine of compensation”, to explain recovery (specifically of movement) based on multiple cerebral representations, any one of which might take over a particular function following injury, leading to clinical recovery.

Jackson’s systems-level thinking has now largely given way to neural network models to explain neurological function and dysfunction. For example, the neurobiological substrates of memory function have been conceptualised in terms of the engram, an historical idea that, after initially seeming a dead end, has attracted much experimental attention in recent times. This conceptualisation is now explored to see if it might be applicable to the memory dysfunction of TGA, TEA, and functional amnesia.

## 3. Engram

The engram conception was first postulated by Richard Wolfgang Semon (1857–1918), a German zoologist working as a private scholar in Munich [21,22]. In his book *Die Mneme*, published in 1904 (first English translation, *The Mneme*, in 1921), Semon defined the engram as “the enduring though primarily latent modification of the irritable substance produced by a stimulus”. The process giving rise to new engrams Semon termed “engraphy” and their recall “ecphory”, but he declined to speculate on the neural mechanisms underlying these conceptions.

Semon’s ideas were largely ignored by his contemporaries, and it was not until the early to mid-20th century that they were popularised by the work of Karl Lashley (1890–1958). However, Lashley eventually characterised the search for the engram in terms of *The Hunting of the Snark*, Lewis Carroll’s 1876 nonsense poem, which may stand as a metaphor for the misguided search for a non-existent object [23]. However, in the past ten to fifteen years, experimental work that claims to define the neural substrates of the engram has emerged [24,25,26,27,28,29].

A convenient modern definition of the engram is that of Moscovitch: “the engram or memory trace is the representation of an encoded event or experience. It is not yet a memory but provides the necessary (physical) condition for memories to emerge. Put another way, the engram is permissive or necessary for memory but does not suffice for its materialization. A memory emerges when the engram interacts with retrieval cues or information derived from particular environmental conditions, a process which Semon termed ‘ecphory’. The product of this retrieval-engram interaction is a memory. Without retrieval, there is only the engram, and even its existence is inferred from memory’s emergence” [30]. Tulving likewise noted that, at least in a limited sense, the engram only comes into existence when “ecphorised” (i.e., activated) [31]. Although there are conceptual objections to the notion of “the engram” [32], for the purposes of this work, “engram” will be considered to denote a distributed ensemble of neurones that are active during memory formation and recall, and for which there is robust empirical evidence.

Engrams are not only distributed rather than localised but also dynamic rather than static, fixed, and immutable. Engrams may be considered active, latent, or silent, with properties that evolve over time. Active engrams are present and accessible; latent engrams are present but not readily accessible through natural cues; silent engrams are present but inaccessible through natural cues although may be amenable to reactivation through certain experimental manipulations to the latent state. The state of an engram may be interchangeable between these conditions—for example, in relation to social experiences or stressful events [33]. Forgetting may be a consequence of the transformation of engrams from a responsive to an unresponsive state—for example, to external cues. Importantly, silent engrams are envisaged to be dormant rather than erased, and hence not necessarily irrevocably lost to recall.

Initial memory encoding occurs in the hippocampal formation. The dentate gyrus (DG), which has approximately an order of magnitude more cells compared to the upstream entorhinal cortex or the downstream CA3 region of the hippocampus proper, is recognised to have a gating function [34]. By filtering afferent inputs to CA3, the DG enacts a sparse coding scheme that permits overlapping or very similar inputs to the hippocampus to be separated from one another, which is the process of pattern separation, and hence, many different memories may be encoded and stored. Adult neurogenesis in the DG is also required for new memory formation, maintaining memory capacity by keeping the gate open, perhaps by reducing synaptic potentiation and eliminating interference to new knowledge from old memories [28]. The recurrent connections of CA3 pyramidal neurones that form an autoassociative network are thought to be important for pattern completion, and hence for the recall of existing memories. Information is then relayed to cortical sites via hippocampal CA1 and the subiculum, with engram ensembles for a single memory being distributed across multiple brain regions [35]. Mechanisms of neuronal plasticity that may be relevant to engram function include enhanced connectivity between the cells composing engram ensembles, through Hebbian-type coordination of presynaptic and postsynaptic activity producing changes in synaptic strengths through the mechanism of long-term potentiation, through morphological changes in dendritic spine density, and through epigenetic changes in specific neurones [29]. Circuit remodelling may underpin forgetting and the transition from episodic to semantic memory and the relative importance of enduring hippocampal or cortical roles [26,28]. Of course, some types of memories (e.g., semantic memory) may be stored and recalled, or even formed (e.g., procedural memory, Pavlovian conditioning), without the involvement of the hippocampus. Engram cell ensembles located in extra-hippocampal brain areas [35] might account for these observations.

The neural substrates of the engram are presumed to be both cellular and synaptic. Functional connectivity between engram cell ensembles is the basis for retained memory. Encoding also results in augmentation of synaptic strength and dendritic spine density, whereas silent engram cells lack synaptic strength and a learning-induced increase in dendritic spine density. Some of the molecular mechanisms that may contribute to engram switching between responsive and unresponsive states have been defined experimentally. For example, overexpression of a particular kinase (α-p-21-activated kinase 1, or PAK1) has been found to convert silent engram cells to the active state with associated morphological changes, specifically an increase in spine density in the engram cells [36]. Activation of the hippocampal activity of the multifunctional Ras-related C3 botulinum toxin substrate 1 (Rac1) protein has been shown to drive latent engrams to silence, whereas suppression of Rac1 activity—for example, through direct Rac1 inhibition—switches silent engrams to the latent state. These effects may be mediated through modification of synaptic connectivity [33].

From the clinical perspective, one of the most intriguing aspects to emerge from engram research is the idea that so-called “lost memories” might be recovered, or, in other words, that latent or silent engrams might be reactivated. This prospect has obvious implications for the treatment of amnesic disorders.

## 4. “Engram-Centric” Approach to Amnesia: AD as Exemplar

Is the engram conception applicable to amnesic disorders generally, and specifically to acute-onset amnesias? J.M. Nielsen’s work from 1958 entitled *Memory and amnesia* appealed repeatedly to the idea of engrams to explain memory impairment in various clinical situations [37] and may have been the earliest work to do so.

Although not an acute-onset amnesia, Alzheimer’s disease (AD) has already been analysed in terms of what may be termed “engram-centric” models [28,38] (“engrammatic” might also suffice) and hence may act as an exemplar. AD typically presents with deficits in episodic or autobiographical memory, functions thought to be mediated initially through processing in the hippocampal DG (in common parlance, “short-term memory”) followed by associatively learned back projections to the neocortex that allow subsequent retrieval of information (“long-term memory”). In mouse models of early AD, memory retrieval by activating silent engram cells was demonstrated to be feasible [39]. An “engram-centric” model of the amnesia of AD has been presented, suggesting that in its early stages, the disorder is one of memory retrieval rather than memory storage and hence possibly amenable to treatment by means of the activation of engram cells [38].

Study of engrams in the AD brain is difficult, but it may be that the typical pathological changes seen in AD, the deposition of beta-amyloid and hyperphosphorylation of tau, might alter the connectivity of engram neurones. Changes in hippocampal DG neurogenesis might also contribute: ageing-related decline or dysregulation through changed expression of genes encoding amyloid precursor protein (APP), presenilin-1 (PS1), or apolipoprotein E (ApoE) may result in fewer immature neurones being recruited into the engram ensemble [28]. Whether any of these might be viable targets for treatment of the amnesia of AD remains to be shown.

## 5. “Engram-Centric” Approach to Acute-Onset Amnesias

In light of the example of AD, the possible implications of an “engram-centric” approach to selected acute-onset amnesic disorders are now explored (see Table 1 for clinical details and differences between the selected conditions), beginning with TGA since it is the most commonly encountered of these conditions.

### 5.1. Transient Global Amnesia

In his publication on “Single episode of confusion with amnesia” presented to the New York Neurological Society [1], Morris Bender’s first reference was to Nielsen’s monograph *Memory and amnesia* [37]. Despite Nielsen’s evident familiarity with the many clinical presentations of amnesia, his book contained no case suggestive of TGA, indicating that this symptom pattern, now so obvious clinically, was not yet widely recognised. Yet, a pervasive theme in Nielsen’s book was that of the engram. Is this conception applicable specifically to TGA?

The deficit in episodic memory in TGA is characterised by a dense anterograde amnesia, often manifested by a striking tendency to ask the same questions repeatedly. Whilst there is also retrograde amnesia of variable extent and some executive functions may be diminished, anterograde amnesia for episodic autobiographical memory is the principal finding. In AD, episodic autobiographical memory is most often the first and most evidently affected cognitive domain, and hence the “engram-centric” models of AD mentioned earlier [28,38] might possibly be transferable to TGA in modified form.

The hypothesis proposed here is that the distributed ensembles of engram neurones normally activated during the formation of new memories fail to become appropriately activated in an episode of TGA. Hence, no new engrams (i.e., ensembles of activated engram cells) are created, there is no pattern separation at the DG level, no new memories are encoded, and there is dense anterograde amnesia. In the historical terminology of Semon, there is a failure of engraphy. The neurobehavioural consequence is that, after recovery from the acute TGA episode, patients have an “amnestic gap” usually covering a period of some hours (as experienced by Mr. H. [1]).

Additionally, failure of CA3 autoassociation impairs pattern completion with a failure to recall existing memories, the engrams for which are now latent or silent, hence the variable retrograde amnesia. In Semon’s terminology, there is an impairment of ecphory.

**Figure 1:**
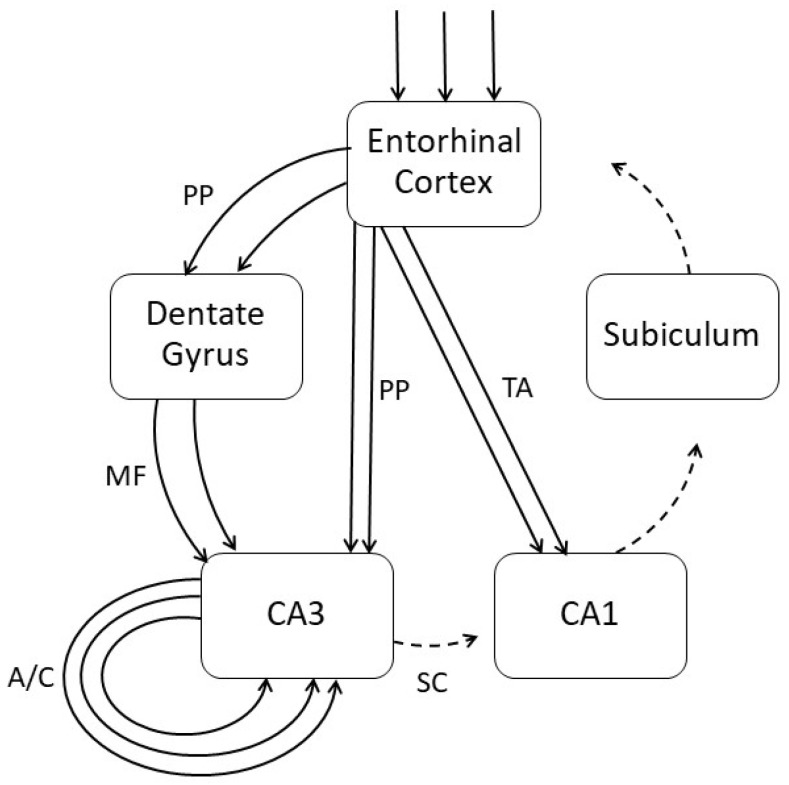
Schematic illustrating the proposed TGA and TEA pathogenesis. Spreading depolarization in the entorhinal cortex transmits through the hippocampal formation, causing excessive positive feedback in the CA3 associative/commissural loop with failure of onward synaptic transmission to the CA1 and neocortex, thus disrupting engram formation. PP = perforant path, MF = mossy fibres, TA = temporoammonic pathway, A/C = associative/commissural loop, SC = Schaffer collaterals.

The most widely accepted mechanistic model of TGA holds that the process of spreading depolarization (SD), sometimes known as cortical spreading depression, underpins TGA [40]. The cascade of biochemical and biophysical changes consequent on SD causes prolonged neuronal membrane depolarization and refractoriness to neuronal impulse and synaptic transmission in hippocampal CA3. This catastrophic degradation of the CA3 autoassociative network results in temporary functional ablation of the whole hippocampal neural network (it closes the DG gate). The consequent failure of feedforward excitation of CA1 from CA3, and hence also of back projections to the neocortex from CA1, may explain both the anterograde amnesia and the failure to retrieve previously learned information, hence contributing to the retrograde amnesia. This “engram-centric” model for TGA pathophysiology is easily reconciled with a recently proposed neural network “attractor hypothesis” model of TGA (Figure 1), based on excessive positive feedback in CA3 autoassociative connections [41].

Another interesting possibility of the “engram-centric” model of TGA is that it may help to explain one of the demographic factors observed to influence the likelihood of having TGA, namely, the age distribution of cases. TGA is a condition of mid-life: most large studies show a mean age of first occurrence in the early 60s, with cases occurring under 40 years of age being distinctly rare. Perhaps failing adult DG neurogenesis, a process that may be important in maintaining memory capacity, may render individuals increasingly vulnerable to the recognised precipitants of TGA with increasing age. The rarity of TGA in older individuals over 80 years may perhaps relate to lower exposure to the most common precipitating factors: emotional stress and physical effort.

As TGA is a self-limiting, and apparently self-reversing, event with no major cognitive sequelae other than the amnestic gap, there would seem to be little indication for experimental therapies aimed at activating latent or silent engrams in this condition, since the expectation is that there are none, corresponding to the time period of the amnestic gap.

### 5.2. Transient Epileptic Amnesia

Whilst Fisher and Adams suspected TGA to be “a special type of focal cerebral seizure” [4], the duration of TGA episodes and the low recurrence rate have long been recognised as atypical for epileptic seizures. Moreover, the ictal EEG is usually normal (as in the case of Mr. H. [1]). Furthermore, amnesic seizures, first described by Hughlings Jackson [13], have now been distinguished from TGA as TEA. However, the exact relationship of TGA and TEA has yet to be defined. The observation that occasional patients with TGA later develop typical episodes of TEA, and the possible increased long-term risk of epilepsy in patients with TGA [42,43], may suggest some shared pathophysiology.

An “engram-centric” view of TEA may therefore overlap with that suggested for TGA, with seizure-related failure to form new engrams. SD may be implicated as a pathogenetic mechanism in epileptic seizures as well as in TGA, rendering hippocampal neurones refractory to impulse transmission (Figure 1). However, the partial recall of episodes noted in some TEA patients [17] suggests that the formation of engrams may occur, some of which remain active (“remembering not being able to remember”), whereas others may be latent or silent. In the historical terminology of Semon, there is both a partial failure of engraphy and an impairment of ecphory. However, because of the brevity of the amnesic episodes in TEA, any attempt to reactivate latent or silent engrams by experimental means might not be deemed worthwhile, over and above the treatment of the underlying epileptic seizures with anti-seizure medications, which often results in remission of TEA.

### 5.3. Functional Amnesia

The occasional recovery of “lost” memories, either partial or total, in patients with functional amnesia suggests that an “engram-centric” model may be more readily applicable here than in TGA or TEA. Such recovery implies that latent or, more likely, silent engrams are present, the deficit being one of retrieval or reactivation of previously encoded and consolidated memory. Indeed, it has previously been demonstrated in the experimental context of contextual fear conditioning that silent memory engrams may be the basis for retrograde amnesia induced by a protein synthesis inhibitor (anisomycin) [36]. Hence, in the historical terminology of Semon, engraphy has occurred in functional amnesia (and indeed persists, as evidenced by the ability to learn new information), but there is a failure of ecphory. This is consistent with the idea that brain tissue is non-functioning rather than destroyed (structural brain imaging is usually normal in functional amnesia patients). A possible mechanism relates to inhibitory engrams, one proposed role for which is to keep memory from interfering with other memory traces [28]. This “engram-centric” model for functional amnesia pathophysiology is thus easily reconciled with a recently proposed neural network “catastrophic forgetting hypothesis” or “catastrophic interference hypothesis” model of functional amnesia [20].

In addition to addressing any psychological factors that might be operating in the onset and maintenance of functional amnesia, experimental therapeutic manipulations aimed at converting latent or silent engram forms to the active state might not only be feasible but also clinically useful in this condition since the symptoms may be persistent over months or years, with important consequences for clinical and social recovery. However, cases of functional amnesia are relatively rare and somewhat clinically heterogeneous [19], making recruitment for appropriately randomised controlled clinical trials problematic. As a proxy or surrogate outcome measure, functional imaging studies might be used since these have suggested hypoactivation in prefrontal cortex and hippocampal and para-hippocampal structures in functional amnesia that returns to normal with symptom resolution [44].

## 6. Discussion

The “engram-centric” conceptualisation of selected acute-onset amnesias presented here may be summarised as follows, firstly in the original terminology of Semon, and secondly in an attempted neurobiological definition:

- TGA: failed engraphy and ecphory; failure to activate any new engram neuronal ensembles, resulting in anterograde amnesia, and impaired activation of existing engram neuronal ensembles, resulting in variable retrograde amnesia.
- TEA: partial failure of both engraphy and ecphory; some new engram ensembles are activated, but not all remain in an active state—some are latent and some are silent.
- Functional amnesia: failed ecphory resulting in a dense retrograde amnesia, but with engraphy intact such that anterograde memory is preserved; pre-existing engram neuronal ensembles are latent or silent, whilst new ensembles may be activated.

Whilst this overview may be too simplistic, nevertheless it suggests that experimental manipulations to enhance or speed recovery would most likely be of benefit in the context of functional amnesia, if it is accepted that these individuals harbour latent or silent engrams.

The lack of compelling animal models for TGA and functional amnesia poses a difficulty for pursuing these possibilities experimentally. For example, there are no data answering the question as to how or whether neural plasticity contributes to the recovery process in these conditions. Furthermore, the animal experimental evidence on engrams accrued thus far has focused on the neural underpinnings of conditioning, such as eye-blink and fear reactions. If memory is understood as the retention of knowledge acquired, conditioned reactions are not a form of knowledge. The experimental animals have come to respond in certain ways to certain stimuli, and these responses may be manipulated experimentally, but the animals have not remembered anything and they have not learned the way to do anything. Indeed, these are non-cognitive abilities. Thus, just as it has been argued that Lashley’s spatial memory (maze-learning) task was an “inappropriate behavioural test to probe an engram” [24], so the studies purporting to characterise memory engrams may also be inappropriate in that conceptually they are not studies of memory at all [32,45]. Simple associative tasks may not be valid models to study the intricacies of the more complex declarative system. The fact that declarative memory and skill learning (procedural memory) are dissociable, as shown in patient HM and possibly also in TGA, adds to the necessity for such a demonstration.

The proposed “engram-centric” models of three acute-onset amnesias presented here help to conceptualise these disorders in terms of distributed and dynamic neuronal ensembles that, because of their plasticity, can evolve over time, thus accounting for the clinical characteristics of these conditions. The models may also have implications for treatment in terms of reactivating latent or silent engrams should safe methods to effect such changes become available. If so, any proposed manipulation of human memory will necessitate careful ethical scrutiny [46].

## Figures and Tables

**Table 1:** Comparison of typical features of transient global amnesia (TGA), transient epileptic amnesia (TEA), and functional amnesia.

Clinical Features	TGA	TEA	Functional Amnesia
Anterograde amnesia during episode?	Yes	Yes	No
Focal neurological deficits during episode?	No	No	No
Aura, automatisms?	No	Yes	No
Symptom duration	<24 h	Usually < 1 h	Variable
Recurrence rate	Low	High	Varied
Triggers	Emotional stress, physical exertion	On waking	Emotional stress, minor head injury
Response to anti-seizure medications	Nil	Usually prompt	Nil
EEG abnormalities during attack?	No	Often	No

## Data Availability

No new data were created or analyzed in this study. Data sharing is not applicable to this article.
